# Knocking down USP39 Inhibits the Growth and Metastasis of Non-Small-Cell Lung Cancer Cells through Activating the p53 Pathway

**DOI:** 10.3390/ijms21238949

**Published:** 2020-11-25

**Authors:** Jiahui Yuan, Gongye Zhang, Xiaomei Li, Qiujuan Ma, Weipeng Cheng, Weiwei Wang, Bing Zhang, Tianhui Hu, Gang Song

**Affiliations:** 1 Cancer Research Center, School of Medicine, Xiamen University, Xiamen 361102, China; 18332921856@163.com (J.Y.); zgypiano@163.com (G.Z.); xmlee0104@163.com (X.L.); MQJHXF@163.com (Q.M.); 24520181154742@stu.xmu.edu.cn (W.C.); wangweiwei_18@163.com (W.W.); thu@xmu.edu.cn (T.H.); 2 Department of Basic Medicine, School of Medicine, Xiamen University, Xiamen 361102, China; cristal66@xmu.edu.cn

**Keywords:** USP39, proliferation, growth, metastasis, p53 pathway

## Abstract

Ubiquitin-specific protease 39 (USP39), a member of the deubiquitinating enzyme family, has been reported to participate in cytokinesis and metastasis. Previous studies determined that USP39 functions as an oncogenic factor in various types of cancer. Here, we reported that USP39 is frequently overexpressed in human lung cancer tissues and non-small-cell lung cancer (NSCLC) cell lines. USP39 knockdown inhibited the proliferation and colony formation of A549 and HCC827 cells and decreased tumorigenic potential in nude mice. Specifically, knocking down USP39 resulted in cell cycle arrest at G2/M and subsequent apoptosis through the activation of the p53 pathway, including upregulation of p21, cleaved-cas3, cleaved-cas9 and downregulation of CDC2 and CycinB1. Moreover, USP39 knockdown significantly inhibited migration and invasion of A549 and HCC827 cells, also via activation of the p53 pathway, and downregulation of MMP2 and MMP9. Importantly, we verified these results in metastasis models in vivo. Collectively, these results not only establish that USP39 functions as an oncogene in lung cancer, but reveal that USP39 has an essential role in regulating cell proliferation and metastasis via activation of the p53 pathway.

## 1. Introduction

Lung cancer is a leading cause of cancer-related death worldwide [1]. This tumor is divided into two major categories: Small-cell lung cancer (SCLC) and non-small-cell lung cancer (NSCLC), accounting for approximately 15%, and 85% of cases, respectively [2,3]. Although there are various effective treatments available for NSCLC, including surgery, radiation therapy, chemotherapy and the new clinical interventions of immunotherapy and molecular targeted cancer therapy, the survival rate still languishes at only 15% within five years of treatment [4,5]. Local recurrence and metastasis are the most common reasons for poor survival, and contribute to the poor prognosis [6]. Therefore, it is imperative to better clarify the underlying new molecular mechanism of NSCLC, which are essential for guiding the discovery of new drugs and treatment approaches.

Ubiquitin-specific protease 39 is a member of the deubiquitinating enzyme family. It contains an N-terminal RS domain, a conservative central zinc finger domain and two C-terminal ubiquitin hydrolase domains [7]. However, despite belonging to this family, USP39 has no activity as a deubiquitinating enzyme because of the absence of two critical amino acid residues, namely cysteine and histidine [8,9].

Previous reports had identified that USP39 is crucial for the process of cytokinesis and pre-mRNA splicing [10,11,12,13,14]. Moreover, recent studies indicated that knocking down USP39 promoted cell proliferation in different types of cancers [15,16,17,18]. Zhifeng Lin et al. demonstrated that USP39 deubiquitinase is significantly overexpressed in lung cancer and promotes the growth of A549 cells and 95D cells in vitro [19]. Recent report identify USP39, serves as a novel regulator of CHK2, regulates DNA damage response and chemo-radiation resistance in A549 cells [20]. However, Julia M. Fraile et al. discovered that USP39 silencing has no effect on the viability and tumorigenic potential of A549 cells, because A549 cells is a KRAS-independent lung cancer cells [21]. These data illustrated that USP39 plays an important role in the proliferation and chemo-radiation resistance of lung cancer cells, but the special function and mechanisms need to be further verified and explored. Therefore, the main purpose of this paper is to verify and confirm the function of USP39 in lung cancer cells and to explore novel molecular mechanisms. In the present study, we examined the expression of USP39 in lung cancer and found that it was significantly upregulated in lung cancer tissues compared with normal lung tissues. Furthermore, we demonstrated that knocking down USP39 inhibited tumorigenesis in vivo and in vitro, induced cell cycle arrest, triggered cell apoptosis, and prevented metastasis of A549 and HCC827 cells in vivo and in vitro. Most importantly, the depletion of USP39 was found to result in the activation of the p53 pathway and to promote the expression of the p53 targets p21, CDC2, cyclinB1, MMP2 and MMP9. Based on these results, we propose that USP39 is a potential oncogene in NSCLC.

## 2. Results

### 2.1. Expression of USP39 Is Significantly Increased in Human Lung Cancer Tissues and NSCLC Cell Lines

**Figure 1:**
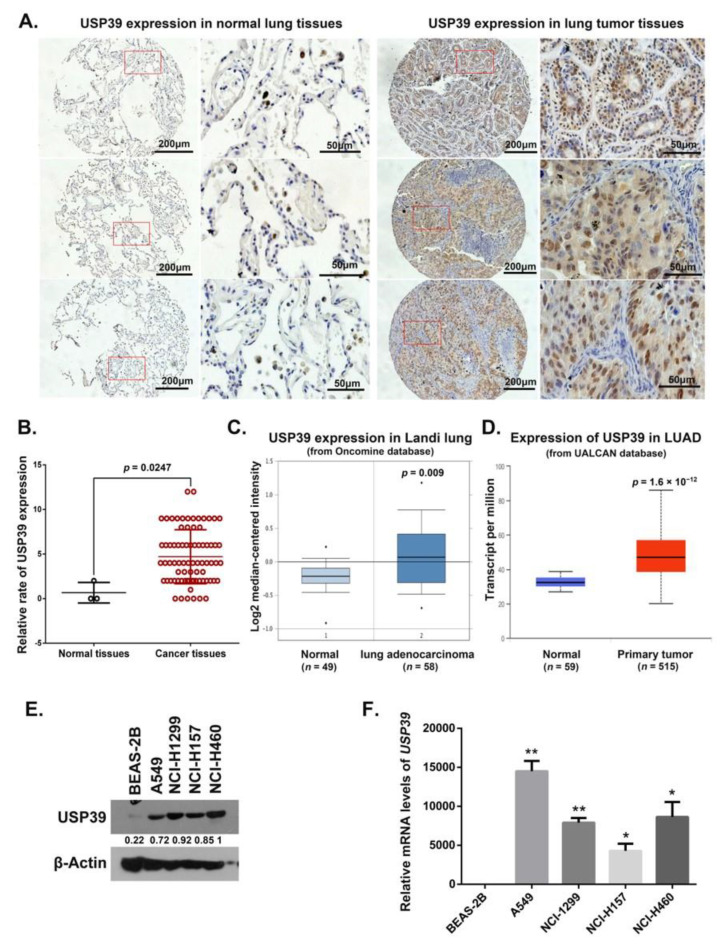
USP39 expression in lung cancer tissues and lung cancer cell lines. (**A**) Representative images of USP39 immunohistochemical staining in normal lung tissues (left) and NSCLC tissues (right) were shown. Magnification 40× and 200×. (**B**) Quantitative analysis of IHC results showed that USP39 protein level was overexpressed in lung cancer tissues. (*n* = 3 in normal group and *n* = 77 in cancer group, * *p* = 0.0247). (**C**,**D**) Gene expression data from Oncomine database and GEPIA database showed that *USP39* mRNA level was overexpressed in human lung cancer. (**E**,**F**) The expression of UP39 was analyzed by Western blot and Real-time PCR in human normal lung cell BEAS-2B and various NSCLC cell lines: A549, NCI-H1299, NCI-H157 and NCI-H460 (* *p <* 0.05, ** *p <* 0.01).

To analyze USP39 expression in lung cancer tissues, using immunohistochemistry we first determined the levels of expression of the USP39 protein in 80 samples from lung cancer patients (comprising 3 normal tissues and 77 lung cancer tissues). Stronger staining of USP39 was observed in the non-small cell lung cancer (NSCLC) tissues, and mainly located in the nucleus (Figure 1A). The frequency of USP39 positivity was higher in clinical NSCLC, compared to the normal lung tissues (*p* = 0.0247) (Figure 1B). We also examined the relationship between the level of USP39 expression and the clinicopathological characteristics of patients from whom the tissue samples were derived. However, no correlations between the levels of USP39 expression with sex, Tumor-Node-Metastasis (TNM) stage, or lymph node invasion were present (Table S1). In addition, we analyzed the gene expression of *USP39* in lung cancer samples using the Oncomine database (https://www.oncomine.org) and GEPIA database (http://gepia.cancer-pku.cn). The results showed that the USP39 mRNA level was significantly increased in lung cancer samples (Figure 1C,D). Next, we assessed USP39 expression in normal bronchial epithelial cells (BEAS-2B) and NSCLC cell lines (A549, NCI-H1299, NCI-H157 and NCI-H460). As depicted in Figure 1E,F, USP39 expression was significantly higher in NSCLC cell lines than in BEAS-2B cells (* *p* < 0.05, ** *p* < 0.01). These results suggest that USP39 may serve as a potential molecular target in lung cancer patients.

### 2.2. Knocking Down USP39 Inhibits A549 Cell Growth in Vivo and In Vitro

**Figure 2:**
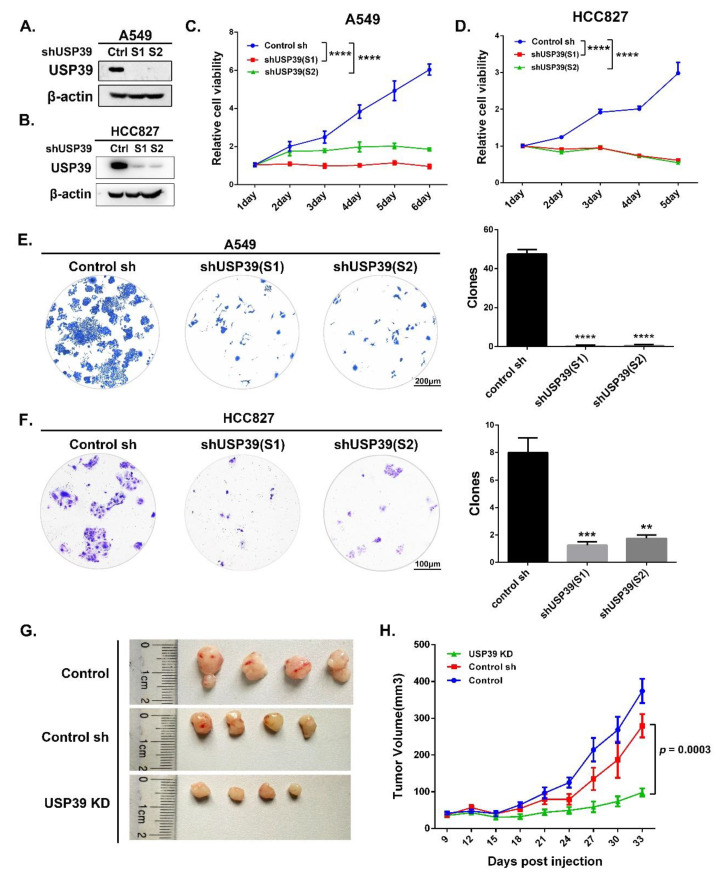
Knocking down USP39 suppresses lung cancer cell proliferation in vivo and vitro. (**A**,**B**) Identification of knockdown efficiency in A549 and HCC827 cells by western blot assay. (**C**,**D**) Stable USP39 knockdown cell lines were plated into 96-well plates and cell viability was examined every 24 h by MTT assay, lasting for 5–6 days (**** *p <* 0.0001, *n* = 4). (**E**,**F**) Meanwhile, Colonies (>50 μM) were counted 10–12 days in A549 and HCC827 cells after transfected by lentivirus mediated USP39 shRNA or control sh groups (** *p <* 0.01, *** *p <* 0.001 and **** *p <* 0.0001). (**G**,**H**) Xenograft tumors were by injection of A549 cells stably suppressing USP39 compared with the control and control sh groups (*n* = 4). Representative images of xenograft tumor were shown. Tumor mass volume was every 3 days after 9 days of injection (*** *p* = 0.0003).

To investigate the roles of USP39 in lung cancer, we generated USP39 shRNAs (control, S1 and S2) lentiviruses and established A549 and HCC827 cell lines stably expressing these shRNAs. As shown in Figure 2A,B, Western blotting analysis revealed that the USP39 protein levels were significantly downregulated in both the shUSP39(S1) and shUSP39(S2) groups compared with the control sh group. Thus, it was demonstrated that shRNAs targeting USP39 exerted significant knockdown effects on USP39 expression. To determine the role of USP39 expression on lung cancer cell viability, MTT assays and colony formation assays were performed on A549 and HCC827 cells. As shown in Figure 2C–F, knocking down USP39 significantly inhibited cell growth (** *p <* 0.01, *** *p <* 0.001, **** *p <* 0.0001). We then further examined the functional consequences of inhibiting USP39 on the growth of A549 cells in vivo. Xenograft tumors of the USP39 KD group exhibited smaller tumor volumes compared with tumors of control and control sh groups (Figure 2G,H). Together, these data indicate that USP39 functions as a tumor promotor and positively regulates lung tumor growth.

### 2.3. Knocking Down USP39 Inhibits the G2/M Cell Cycle Transition and Induces Apoptosis

**Figure 3:**
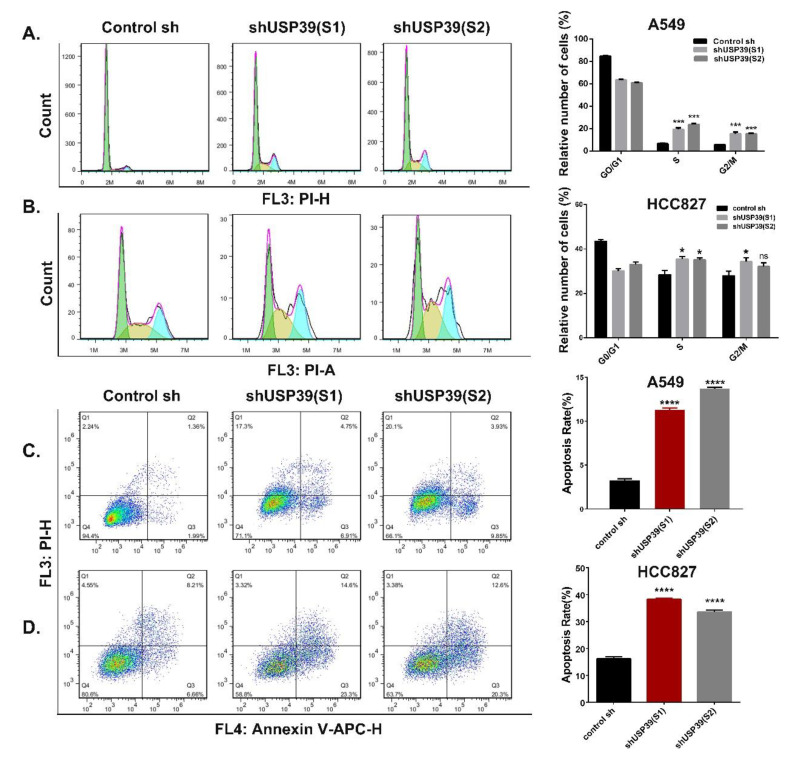
Knocking down USP39 induces cell cycle arrest and apoptosis. (**A**,**B**) Cell cycle distributions of A549 and HCC827 stable cell lines USP39 knockdown and the control cells were examined using flow cytometry. Percentages of cells in each phase are indicated (* *p* < 0.05 and *** *p* < 0.001). (**C**,**D**) Representative images and quantitative analysis of PI/Annexin V-APC staining results by using flow cytometry in A549 and HCC827 cells (*** *p* < 0.001 and **** *p* < 0.0001).

**Figure 4:**
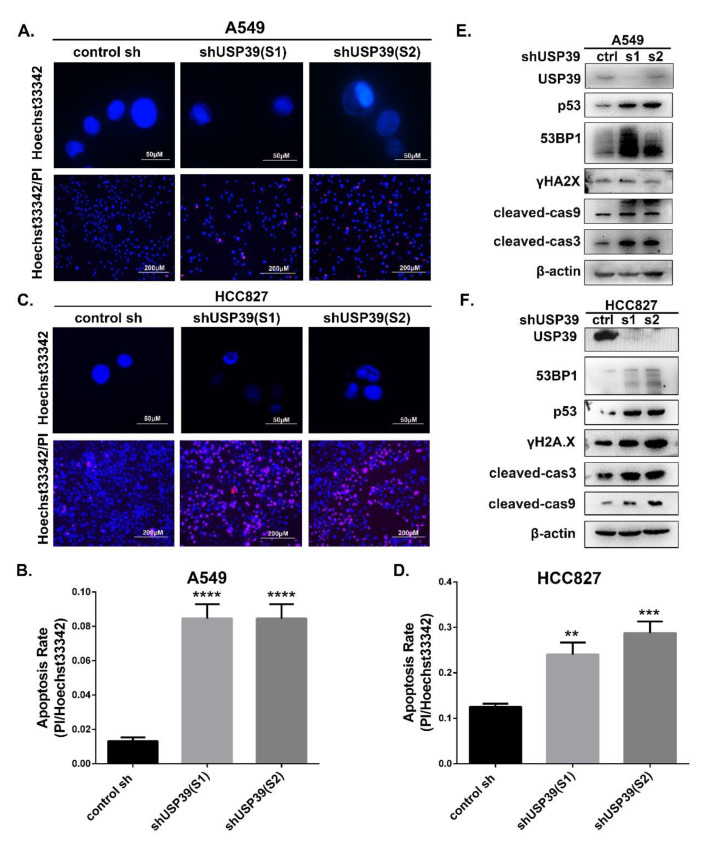
Regulation of apoptotic markers in A548 and HCC827 cells by USP39 knockdown. (**A**–**D**) Representative fluorescence micrographs of Hoechst33324 (blue)/PI (red) of A549 and HCC827 cells, and quantitative analysis of the positive ration of apoptosis showed that knockdown of USP39 induced apoptosis (** *p* < 0.01, *** *p* < 0.001 and **** *p* < 0.001). Meanwhile, Representative fluorescence micrographs of Hoechst33324 (blue) of A549 cells. We detected that chromatin present concentrated partly and some vacuoles in USP39 knockdown groups. The nucleus breaks down into fragments, producing apoptotic bodies in USP39 knockdown groups. (**E**,**F**) Western blot analysis of apoptotic markers (cleaved-cas3 and cleaved-cas9) and DNA damage markers (p53, 53BP1 and γH2AX) expressions in A549 and HCC827 cells.

To elucidate the molecular mechanisms by which USP39 knockdown inhibits cell proliferation, we next investigated its effect on cell cycle distribution and cell apoptosis. Cell lines in which USP39 was stably knocked down exhibited a decrease in G1 phase cells and a concomitant increase in S phase cells and G2/M phase cells compared with control cell lines (Figure 3A,B; * *p* < 0.05, *** *p* < 0.001). Interestingly, during culture of the USP39 KD cell lines, we observed changes in cellular morphology; the cells became smaller, rounder and wizened, which is a sign of cell apoptosis. Therefore, we assessed the effect of USP39 on cell apoptosis by a PI/Annexin V-APC staining-based flow cytometric method in A549 and HCC827 cells. The proportion of apoptotic cells in USP39 KD lines was higher than in the controls (Figure 3C,D, *** *p* < 0.001, **** *p* < 0.001). We then performed Hoechst33342 staining and Hoechst33342/PI double staining assays to evaluate cell apoptosis. As described in Figure 4A–D, apoptotic cells are stained red by PI, revealing that the frequency of apoptotic cells in the USP39 KD groups was significantly higher than in the control group (*** *p* < 0.001). In Figure 4A,C, the nucleus of USP39 KD cells presented some obvious changes of morphology on staining by Hoechst33342, namely rippled or creased appearance, with some chromatin condensed and marginalized, and much debris. In order to further demonstrate the mechanisms of apoptosis induced by USP39 knockdown, the levels of cleaved cas3 and cleaved cas9 were increased in response to USP39 knockdown (Figure 4E,F). In addition, we observed that the expression of DNA damage markers (p53, 53BP1 and γH2AX) was markedly increased in cells which USP39 had been knocked down (Figure 4E,F). Collectively, our data suggest that knocking down USP39 induces cell cycle arrest at S phase and G2/M phase, and results in apoptosis, and that the mechanism of apoptosis induced by USP39 knockdown may be related to DNA damage.

### 2.4. Knockdown of USP39 Inhibits Metastatic of Lung Cancer Cells In Vivo and In Vitro

**Figure 5:**
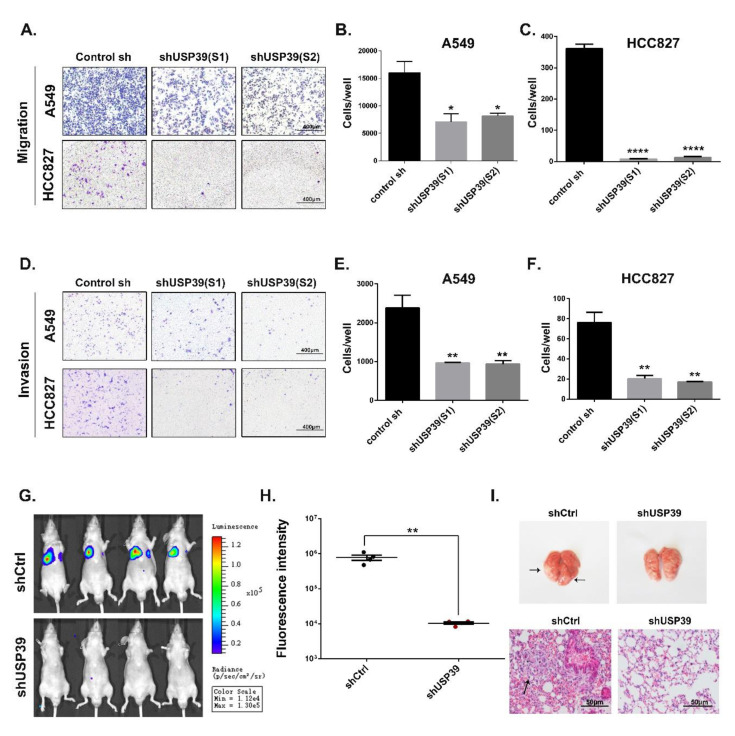
Knocking down USP39 inhibits metastasis of in vivo and in vitro. (**A**–**C**) cell migration and (**D**–**F**) invasion were determined by transwell assay (non-coated version of the Matrigel for migration, coated version for invasion) in A549 and HCC827 cells transfected by shRNA USP39 and control cells (* *p* < 0.05, ** *p* < 0.01 and **** *p* < 0.0001). (**G**,**H**) in vivo metastasis of A549 cells: The A549 cells (stably expressing shCtrl or shUSP39) expressing luciferase were injected by tail vein. The needle mice imaged using non-invasion live animal imaging system. The luminescence images and statistical analysis from both groups was quantitated in 8 weeks after iv (** *p* < 0.01, *n* = 4). (**I**) anatomy images of lung metastases and H&E images of lung sections (metastasis nodules pointed by arrow).

To test the potential contribution of USP39 to the metastatic capacity of A549 and HCC827 cells, in vitro, we performed Matrigel non-coated Transwell migration and Matrigel coated Transwell invasion assays. As shown in Figure 5A–F, USP39 knockdown reduced the cell migration and invasion of both USP39KD cells in comparison to the control cells (* *p* < 0.05, ** *p* < 0.01, **** *p* < 0.0001). Accordingly, we obtained the same results in animal experiments. We cultured luciferase-expressing A549 cells (stably expressing shctrl or shUSP39). The A549 (shctrl) and A549 (shUSP39) cells were then suspended in PBS (2 × 10^7^/mL) and 100 μL of the cell suspension was injected into nude mice via the tail vein. Eight weeks after the injection, the growth of metastatic cells was monitored by a non-invasive imaging technique following luciferin injection. Our results showed that luminescence intensity in the lungs of the shUSP39 group was higher than in the control group (Figure 5G,H, ** *p* < 0.001, *n* = 4). As shown in Figure 5I, microscopy images of lung metastases and H&E images of lung sections demonstrated that the numbers of metastatic nodules in the lungs from the shUSP39 group were significantly fewer compared to the control group. Together, these data demonstrate that USP39 knockdown inhibits the metastasis of lung cancer cells in vivo and in vitro.

### 2.5. Knocking Down USP39 Promotes Activation of p53 Signaling

**Figure 6:**
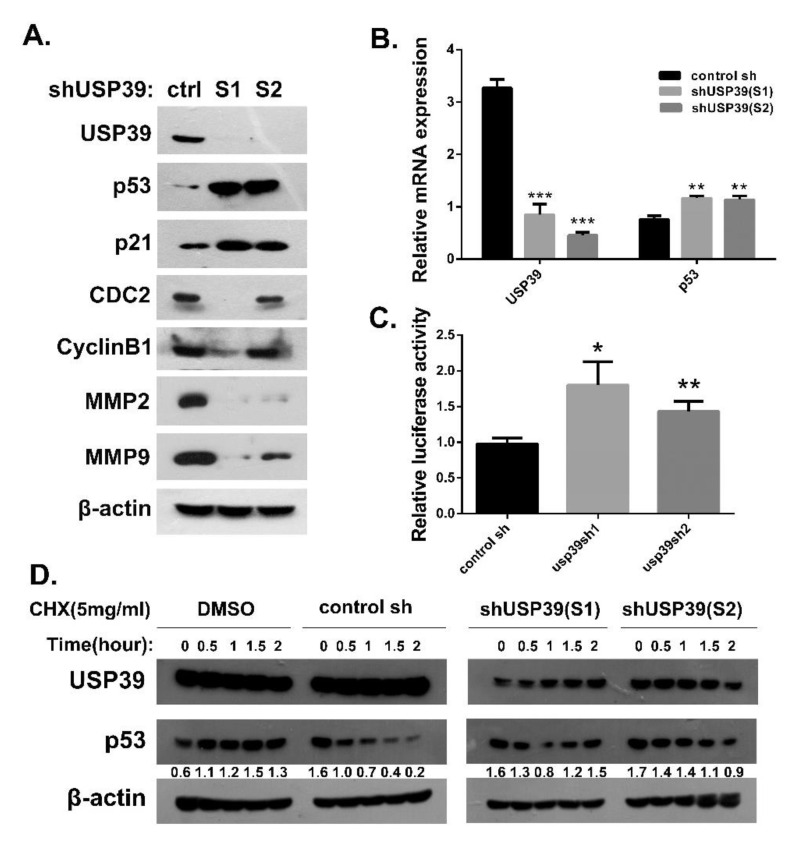
USP39 Knockdown activates p53 and increases the stability of p53. (**A**) Western blot assay was carried out to detect the expression of regulators related to p53/p21 pathway and its downstream molecules including p53, p21, CDC2, cyclinB1, MMP2 and MMP9. (**B**) Suppression of USP39 increases p53 mRNA levels. (**C**) A549 cells stably transfected with shUSP39 were transfected with a p53-responsive reporter (PGL6-TA-TP53) and PRL-TK internal plasmid. The shRNA targeting USP39 increases p53-responsive reporter activity. (**D**) A549 cells were stably transfected with shUSP39 and incubated with cycloheximide (CHX, 5 mg/mL) to inhibit protein synthesis before harvesting. USP39 knockdown increased the half-life of p53. (* *p* < 0.05, ** *p* < 0.01, *** *p* < 0.001).

Our previous data suggested that USP39 might function as a tumor enhancer in human lung cancer. However, the mechanism underlying the effects of USP39 on A549 cells needed to be further explore. The p53 signaling pathway and its regulators play a significant antioncogenic role in lung cancer development and progression. Based on a previous reported by Allende-Vega [22], it was known that USP39 binds multiple key splicing-related proteins participated in splicing reaction of p53 pre-mRNA, and thereby regulates p53 activation. Additionally, based on the AIPuFu database (http://www.aipufu.com/index.html), the finding suggested that USP39 expression is negatively correlated with TP53 in lung adenocarcinoma (shown as Figure S1). Therefore, we tested the effect of USP39 knockdown on p53 activation and its downstream factors in A549 cells. As shown Figure 6B–C, knocking down USP39 caused a significant accumulation of p53, elevated p53-responsive transcriptional reporter activity and an increase in the mRNA and protein level of the p53 target gene p21 (* *p* < 0.05, ** *p* < 0.01). Additionally, USP39 knockdown led to downregulation of CDC2, cyclinB1, MMP2 and MMP9 through activating the p53 pathway (Figure 6A). Meanwhile, we found that USP39 knockdown also activates the p53 pathway, upregulation of p53, p-p53(S15), p21 and BAX in HCC827 cells (Figure S2). To further explore the effect of USP39 on the stability of p53, we treated control cells or cells stably expressing USP39 shRNA with the protein synthesis inhibitor cycloheximide and then determined the half-life of p53. A significant increase in p53 half-life was observed on USP39 knockdown (Figure 5D). Altogether, these results suggest that USP39 knockdown causes a significant accumulation of p53 via regulating both transcriptional levels and post-translational modifications of p53.

**Figure 7:**
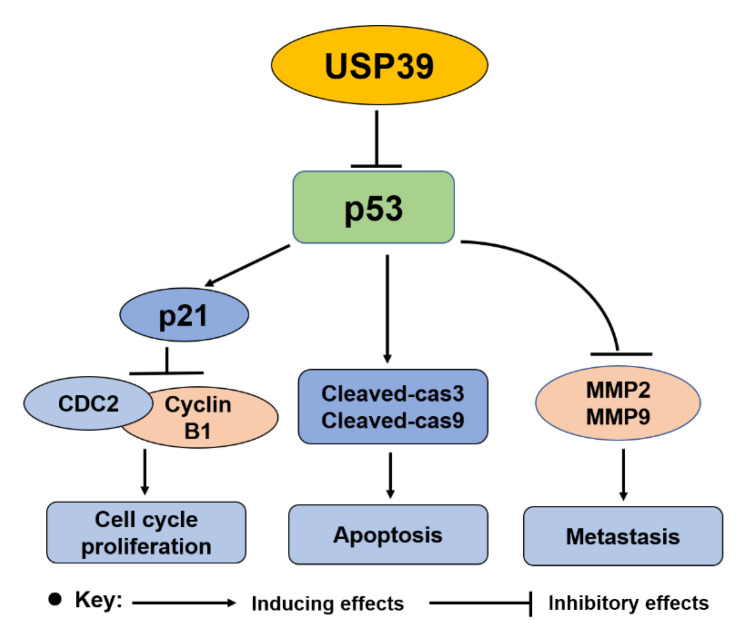
Proposed model illustrating mechanisms of USP39 regulating the proliferation, apoptosis and metastasis of non-small lung cancer cells via regulating the p53 pathway.

Collectively, our findings identified that USP39 as a tumor promotor that plays a vital role in the human lung cancer malignant phenotypes by regulating the p53 pathway (shown as Figure 7). Further studies demonstrated that depletion of USP39 results in an upregulation of p53 through prolonging its half-life and activating its transcriptional activation activity.

## 3. Discussion

Recently, several studies have reported that USP39 is abnormally expressed in various different malignant tumors and may function as a tumor promotor. For example, upregulation of USP39 in hepatocellular carcinoma contributes to the growth of SMMC-7721 cells via regulating the pre-mRNA splicing of FoxM1 [23]. In oral squamous cell carcinoma and medullary thyroid carcinoma, USP39 also functions as an oncogene [24,25]. Our study provides experimental evidence for USP39 upregulation in lung cancer. We further observed that USP39 downregulation inhibits the cell proliferation and migration of A549 and HCC827 cells. Most importantly, we demonstrated that the mechanism of action of USP39 is focused on the p53 pathway, and knocking down USP39 activates the p53 pathway.

This pathway presents a significant tumor suppressor mechanism, which has critical roles in cell cycle arrest, apoptosis, senescence, DNA repair, angiogenesis, autophagy and migration [26,27,28]. Here, we found that knocking down USP39 functions as an anti-oncogene by activating the p53 pathway. First, we observed that depletion of USP39 contributes to abnormal cell cycle distribution by inducing cell cycle arrest at G2/M. Moreover, the underlying mechanism of action of USP39 is partly dependent on activation of the p53 pathway, upregulation of p53 and p21, and downregulation of CDC2 and CyclinB1 [29] in A549 cells. Second, we found that knocking down USP39 induces apoptosis of A549 and HCC827 cells, upregulates cleaved cas3, cleaved cas9 and DNA damage makers (53BP1 and γH2AX) [30]. Third, we demonstrated that USP39 knockdown inhibits cell migration and invasion by upregulating p53 and the downstream proteins MMP2 and MMP9 [31]. In addition, by employing a double luciferase reporter system assay, we found that depletion of USP39 enhances p53-responsive transcriptional reporter activity. Furthermore, USP39 knockdown increased the stability of the p53 protein by prolonging its half-life. In addition, we examined the effect of PFT-α (which is a p53 inhibitor which blocks its transcriptional activity and prevents cells from apoptosis.) on A549 cells to confirm the key role of p53, the WB result demonstrated that exposure to PFT-α (30 or 40 μm/48 h) clearly inhibited the p53 pathway activation, which activated by USP39 knockdown in A549 cells. However, treated with PFT-α reduced the up-regulation of p21 and BAX induced by USP39 knockdown, but had no effect on the down-regulation of cell cycle related proteins (CDK1/CyclinB1 and CDK2/CyclinA2) induced by USP39 knockdown (Figure S3). All the observations thus suggest that the p53 pathway may be a key factor in the tumor promotor function of USP39 in NSCLC, but it is not the only factor.

In summary, our study demonstrated that the oncogenic function of USP39 in NSCLC is crucial of the disease. These observations provide experimental evidence in favor of the exploitation of USP39 as a potential molecular target in NSCLC. A recent study indicated that p53-dependent, miR-1281-mediated USP39 pathway impairs the survival of human osteosarcoma cells under ER stress [32], which suggests that p53 may be an upstream regulator of USP39 and may regulate USP39 expression directly or indirectly. Given the importance of the p53 pathway in the tumor promotor function of USP39, further investigations should address these key questions: (1) How does USP39 regulate p53 and what is the interaction between USP39 and p53? (2) is the oncogenic function of USP39 solely dependent on the p53 pathway, or is another signaling pathway involved?

## 4. Materials and Methods

### 4.1. Lung Cancer Microarray and Immunohistochemistry

Immunohistochemistry was conducted on microarray slides containing 77 lung cancer tissues and 3 normal tissues obtained from Fanpu Biotechnology (Guilin, China). Staining was conducted using rabbit anti-USP39 antibodies (Abcam, Cambridge, MA, USA). Signals were detected using the Vectastain Elite ABC Kit (Ribo Biotechnology, Guangzhou, China). Hematoxylin was used for counterstaining. Based on the previous study [33], the immunoreactivity of USP39 was scored in a semiquantitative way by incorporating both stain intensity and percentage of positive tumor cells. The stain intensity was scored as 0 (no staining), 1 (weak staining), 2 (moderate staining), or 3 (strong staining). The percentage of positive cells was scored as 0 (<12.5%), 1 (12.5~25%), 2 (25–50%), 3 (50–75%), or 4 (>75%). The final score was obtained by multiplying the score of intensity by the score of the percentage of positive cells. Cases with a score of ≤1 were considered negative (−). Cases with a score of >1 were considered positive (+).

### 4.2. Cell Lines and Cell Culture

A549(TP53 WT), HCC827(TP53 WT), NCI-H1299(TP53 NULL), NCI-H157(TP53 WT), NCI-H460(TP53 WT) (http://p53.free.fr/Database/Cancer_cell_lines/NSCLC.html) and 293T cells were purchased from the Cell Bank of Chinese Academy of Sciences (Shanghai, China). Human Bronchial Epithelial Cells (BEAS-2B) cells were obtained from the American Type Culture Collection (ATCC, Cat No CRL-9609), which were cultured in keratinocyte basal medium (KBM-2, USA, Cat No CC-3013) and supplemented with keratinocyte growth medium (KGM-2, USA, Cat No CC-4152). A549, NCI-H1299, NCI-H157, NCI-H460 and HCC827 cell lines were cultured in RPMI-1640 (Gibco, Waltham, MA, USA) with 10% fetal bovine serum (FBS; Gibco; Thermo Fisher Scientific, Inc., Waltham, MA, USA) and 100 IU/mL penicillin and streptomycin. 293T cells were cultured in Dulbecco’s modified Eagle’s medium (DMED; Gibco; Thermo Fisher Scientific, Inc., USA) supplemented with 10% FBS. All cells were maintained at 37 °C in 5% CO_2_.

### 4.3. Lentivirus Production and USP39 Knockdown

To silence the expression of USP39, two plasmids (GV-248-GFP-puro) for human shUSP39 purchased by GENECHEM (Shanghai, China). The shRNA sequences targeting USP39 were as follows: S1, 5′-CGGGTATTGTGGGACTGAA-3′; S2, 5′-TTCCAGACAACTATGAGAT-3′. Lentiviruses were produced in 293T cells as described in a previous report [34,35], A549 and HCC827 cells were incubated in 6-well plates and infected with control sh, shUSP39(S1) and shUSP39(S2) for 48 h. The efficiency of infection was confirmed by the observation of GFP expression with fluorescence microscopy. The efficiency of knockdown was examined by western blot and real-time PCR. (GFP: green fluorescent protein).

### 4.4. Protein Extraction and Western Blot

Protein extraction and western blot assay were performed as previously described [36,37]. Antibodies used are as follows: USP39 (1:2000, Abcam, Cambridge, UK), p53 (DO-1, 1:300, Santa Cruz, CA, USA); p21(12D1, 1:2000, CST); CDC2 (1:1000, Ruiying Biological, Suzhou, China); CyclinB1 (1:1000, Abcam, UK); MMP2 (1:1000, Ruiying Biological, Suzhou, China); MMP9 (1:1000, Ruiying Biological, Suzhou, China); cleaved cas3 and cleaved cas9(1: 1000, Proteintech, Wuhan, China), 53BP1and γH2AX (1:2000, Abcam, UK), p-p53(s15) (1:1000, ABclonal, Woburn, MA, USA), CDK2 (1:1000, Proteintech), CyclinA2 (1:1000, Proteintech), BAX (1:1000, Proteintech), and β-actin (1:40000, Sigma, St. Louis, MO, USA). Additionally, cycloheximide (CHX) and pifithrin-α(PFT-α) were purchased from MedChemExpress.

### 4.5. RNA Extraction and Quantitative PCR Analysis

Total RNA from cells was extracted by using TRIZol reagent (Takara, Shiga, Japan) and reverse-transcribed with the Super-Script First-strand synthesis System for RT-PCR to produce cDNA according to the manufacturer’s protocol (Takara, Japan). SYBR green qPCR Supermix (Takara, Japan) was used for the qPCR reaction, and the expression levels were quantified using the −ΔΔCt method. GAPDH was used as the internal control. The following primer sequences were used:

Homo *USP39* (forward): 5′-TTGGAAGAGGCGAGATAA-3′,

Homo *USP39* (reverse): 5′-AGGAGCATCAATCATCATC-3′;

Homo *Tp53* (forward): 5′-CAGCCAAGTCTGTGACTTGCA-3′,

Homo *Tp53* (reverse): 5′- GTGTGGAATCAACCCACAGCT-3′;

Homo *GAPDH* (forward): 5′-TGCACCACCAACTGCTTAGC-3′,

Homo *GAPDH* (reverse): 5′-GGCATGGACTGTGGTCATGAG-3′.

### 4.6. Cell Proliferation and Colony Formation Assay

Cells were plated into 96-well plates at a density of 2000 cells per well for MTT (*n* = 5). For MTT, cell viability was examined every day after seeding. Briefly, cells were incubated with 20 μL of MTT solution (5 mg/mL, Sigma, USA) at 37 °C for 4 h. The medium was aspirated and 150 μL of dimethyl sulfoxide (DMSO) was added to each well and absorbance at 490 nm was measured on a microplate spectrophotometer.

As to colony formation assay, A549 and HCC827 cells (0.5 × 10^3^/well) were respectively plated into 6-well plates and incubated for 10 days. Cell colonies were washed with PBS, fixed with 4% formaldehyde for 30 min and later stained with 0.1% crystal violet dye for 5 min. Then, the images of colonies were captured by a light microscope. All assays were repeated three times.

### 4.7. Flow Cytometric Assay

For cell cycle analysis, cells were harvested, washed with PBS and fixed in 75% cooling ethanol at 4 °C overnight. Cells were washed with PBS at two times and incubated with RNase A (100 μg/mL, Sigma) for 30 min. Then cells were stained with propidium iodide (PI) solution (50 μg/mL, Sigma) for 15 min at RT and analyzed on flow cytometer. Cell cycle was assessed by staining with PI and analyzed using a flow cytometer.

Cell apoptosis was also measured by FACS analysis using PI/Annexin V-APC Apoptosis Detection Kit (KeyGEN, Nanjing, China). The cells were harvested, washed with PBS, and stained with PI/Annexin V-APC in the dark with 15 min at RT. Finally, the stained cells were detected by a FACS flow cytometer (Beckman, 250 S.Kraemer Boulevard Brea, CA 92821, USA). All experiments were repeated a minimum of three times, and the results was analyzed by FlowJo 7.6 software.

### 4.8. Detection of Apoptosis by Hoechst Staining and Hoechst33342/PI Double Staining

Cells were plated into 24-well plates at a density of 5000 cells per well, washed with PBS and fixed in 4% paraformaldehyde (Sheng gong bio., Shanghai, China) for 30 min. Then, cells were washed with PBS and stained with Hoechst33324 (Ribo bio., Guangzhou, China) for 15 min. The observation of nuclear morphometry was detected on fluorescence microscopy.

In terms of Hoechst33342/PI double staining, cells were plated into 96-well plates at a density of 6000 cells per well, cultured for 24 h, washed with PBS and incubated with 100 μL/well Hoechst33342 (1 μg/mL)/PI (0.5 μg/mL) for 10 min in the dark at room temperature. The cells were captured and counted under a fluorescence microscope. We selected 5 fields from each group randomly, and calculated the apoptosis rate.

### 4.9. Migration and Invasion Assay

For migration and invasion assays, Matrigel-noncoated and Matrigel-coated chambers (BD Biosciences, USA) containing 8 μm pores were used for the assays. Briefly, 5 × 10^4^ (1 × 10^5^ cells for invasion) cells were seeded into the Matrigel-noncoated upper chambers (coated in Matrigel for invasion) in serum-free medium. The lower chamber of the transwell was filled with culture media containing 10% FBS as a chemo-attractant. After the chambers were incubated at 37 °C for 20 h, non-migrated (or non-invaded) cells on the top of the transwell were scraped off with a cotton swab. And then cells successfully translocated were fixed with 4% paraformaldehyde, stained with 0.1% crystal violet, and counted under a light microscope.

### 4.10. In Vivo Tumorigenesis

BALB/c male nude mice were obtained from Shanghai SLAC Bioscience (Shanghai, China). Cell suspensions (2 × 10^6^ cells per 0.1 mL PBS) were injected into the dorsal right flank of mice (*n* = 4) aged 4–6 weeks of age. The longest; (a) and shortest; (b) diameters of tumor masses were measured every 3 days for 33 days and then mice were killed. Tumor volume was calculated as follows: volume (mm^3^) = 0.5 × ab^2^. Care of animals and all animal-related experiments were performed according to the institutional and national guidelines for animal experiments.

### 4.11. In Vivo Metastasis Experiment

The A549 (shctrl) and A549 (shUSP39) cells with luciferase expression were collected and washed with PBS. The cells were resuspended in medium (free serum), at a density of 2 × 10 ^7^/mL. A 100 μL Cell suspension was injected in the tail vein of athymic nude mice. Both the groups had four mouse each. At the completion of this experiment (8 weeks), lungs of 4 mouse from each group were imaged by IVIS ex vivo. Lungs were 4% paraformaldehyde-fixed for 48 h and paraffin embedded for H&E.

### 4.12. Luciferase Reporter Assay

A549 cells stably expressing shUSP39 were seeded into 24-well plate and co-transfected with PGL6-TA-TP53-Luc reporter plasmid and pRL-TK plasmid. After transfection for 30 h, firefly and renilla luciferase were assayed according to manufacturer’s protocol (Promega, Madison, WI, USA). Luciferase activity was expressed as relative light units. Each experiment was repeated in triplicates.

### 4.13. Statistical Analysis

The results of Western blotting, cell proliferation and apoptosis, and migration and invasion were analyzed by using GraphPad Prism software. All values are reported as the mean ± SD, *n* ≥ 3. When significant effects of treatments were indicated, two-sides Student *t* tests, One-way ANOVA analysis and chi-square test were used for two or more groups comparisons, and *p* < 0.05 was considered statistically significant.

## Supplementary Materials

The following are available online at https://www.mdpi.com/1422-0067/21/23/8949/s1.

## References

[1] Siegel R.L., Miller K.D., Jemal A. (2020). Cancer statistics, 2020. CA Cancer J. Clin..

[2] Torre L.A., Siegel R.L., Jemal A. (2016). Lung Cancer Statistics. Lung Cancer and Personalized Medicine.

[3] Herbst R.S., Heymach J.V., Lippman S.M. (2008). Lung cancer. N. Engl. J. Med..

[4] Heist R.S., Engelman J.A. (2012). SnapShot: Non-small cell lung cancer. Cancer Cell.

[5] Spira A., Ettinger D.S. (2004). Multidisciplinary Management of Lung Cancer. N. Engl. J. Med..

[6] Rosenzweig K.E., Gomez J.E. (2017). Concurrent chemotherapy and radiation therapy for inoperable locally advanced non-small-cell lung cancer. J. Clin. Oncol..

[7] Nijman S.M., Luna-Vargas M.P., Velds A., Brummelkamp T.R., Dirac A.M., Sixma T.K., Bernards R. (2005). A genomic and functional inventory of deubiquitinating enzymes. Cell.

[8] Sowa M.E., Bennett E.J., Gygi S.P., Harper J.W. (2009). Defining the Human Deubiquitinating Enzyme Interaction Landscape. Cell.

[9] Fraile J.M., Quesada V., Rodríguez D., Freije J.M.P., Otín C.L. (2011). Deubiquitinases in cancer: New functions and therapeutic options. Oncogene.

[10] Van Leuken R.J., Luna-Vargas M.P., Sixma T.K., Wolthuis R.M., Medema R. (2008). Usp39 is essential for mitotic spindle checkpoint integrity and controls mRNA-levels of aurora B. Cell Cycle.

[11] Hadjivassiliou H., Rosenberg O.S., Guthrie C. (2014). The crystal structure of S. cerevisiae Sad1, a catalytically inactive deubiquitinase that is broadly required for pre-mRNA splicing. RNA.

[12] Chen Z., Gui B., Zhang Y., Xie G., Li W., Liu S., Xu B., Wu C., He L., Yang J. (2017). Identification of a 35S U4/U6.U5 tri-small nuclear ribonucleoprotein (tri-snRNP) complex intermediate in spliceosome assembly. J. Biol. Chem..

[13] Huang Y., Pan X.W., Li L., Chen L., Liu X., Lu J.L., Zhu X.M., Huang H., Yang Q.W., Ye J.Q. (2016). Overexpression of USP39 predicts poor prognosis and promotes tumorigenesis of prostate cancer via promoting EGFR mRNA maturation and transcription elongation. Oncotarget.

[14] Ríos Y., Melmed S., Lin S., Liu N.A. (2011). Zebrafish usp39 Mutation Leads to rb1 mRNA Splicing Defect and Pituitary Lineage Expansion. PLoS Genet..

[15] Dong X., Su H., Jiang F., Li H., Shi G., Fan L. (2018). miR-133a, directly targeted USP39, suppresses cell proliferation and predicts prognosis of gastric cancer. Oncol. Lett..

[16] Jing C., Liu T., Peng H., Yan W., Guo C., Xiong L., Liu A. (2017). USP39, a direct target of microRNA-133a, promotes progression of pancreatic cancer via the AKT pathway. Biochem. Biophys. Res. Commun..

[17] Xing Z., Sun F., He W., Wang Z., Song X., Song X. (2018). Downregulation of ubiquitin-specific peptidase 39 suppresses the proliferation and induces the apoptosis of human colorectal cancer cells. Oncol. Lett..

[18] Xu Y., Zhu M.R., Zhang J.Y., Si G.M., Lv J.J. (2018). Knockdown of ubiquitin-specific peptidase 39 inhibits the malignant progression of human renal cell carcinoma. Mol. Med. Rep..

[19] Lin Z., Xiong L., Lin Q. (2016). Ubiquitin-specific protease 39 is overexpressed in human lung cancer and promotes tumor cell proliferation in vitro. Mol. Cell Biochem..

[20] Wu J., Chen Y., Geng G., Li L., Yin P., Nowsheen S., Li Y., Wu C., Liu J., Zhao F. (2019). USP39 regulates DNA damage response and chemo-radiation resistance by deubiquitinating and stabilizing CHK2. Cancer Lett..

[21] Fraile J.M., Manchado E., Lujambio A., Quesada V., Campos-Iglesias D., Webb T.R., Lowe S.W., López-Otín C., Freije J.M.P. (2017). USP39 Deubiquitinase Is Essential for KRAS Oncogene-driven Cancer. J. Biol. Chem..

[22] Allende-Vega N., Dayal S., Agarwala U., Sparks A., Bourdon J.C., Saville M.K. (2013). p53 is activated in response to disruption of the pre-mRNA splicing machinery. Oncogene.

[23] Yuan X., Sun X., Shi X., Jiang C., Yu D., Zhang W., Ding Y. (2017). USP39 regulates the growth of SMMC-7721 cells via FoxM1. Exp. Ther. Med..

[24] Li K.Y., Zhang J., Jiang L.C., Jiang C., Yu D., Zhang W., Ding Y. (2016). Knockdown of USP39 by lentivirus-mediated RNA interference suppresses the growth of oral squamous cell carcinoma. Cancer Biomark..

[25] An Y., Yang S., Guo K., Ma B., Wang Y. (2015). Reduced USP39 expression inhibits malignant proliferation of medullary thyroid carcinoma in vitro. World J. Surg. Oncol..

[26] Giaccia A.J., Kastan M.B. (1998). The complexity of p53 modulation: Emerging patterns from divergent signals. Genes Dev..

[27] Rufini A., Tucci P.J.F., Celardo I., Melino G. (2013). Senescence and aging: The critical roles of p53. Oncogene.

[28] Reed S.M., Quelle D.E. (2014). p53 Acetylation: Regulation and Consequences. Cancers.

[29] Fragkos M., Jurvansuu J., Beard P. (2009). H2AX is required for cell cycle arrest via the p53/p21 pathway. Mol. Cell. Biol..

[30] Lu Z., Miao Y., Muhammad I., Tian E., Hu W., Wang J., Wang B., Wang B., Li J. (2017). Colistin-induced autophagy and apoptosis involves the JNK-Bcl2-Bax signaling pathway and JNK-p53-ROS positive feedback loop in PC-12 cells. Chem. Biol. Interact..

[31] Yan C., Wang H., Boyd D.D. (2002). ATF3 represses 72-kDa type IV collagenase (MMP-2) expression by antagonizing p53-dependent trans-activation of the collagenase promoter. J. Biol. Chem..

[32] Jiang J., Ma B., Li X., Jin W., Han C., Wang L., Wang H. (2018). MiR-1281, a p53-responsive microRNA, impairs the survival of human osteosarcoma cells upon ER stress via targeting USP39. Am. J. Cancer Res..

[33] Kusinska R.U., Kordek R., Pluciennik E., Bednarek A.K., Piekarski J.H., Potemski P. (2009). Does vimentin help to delineate the so-called ‘basal type breast cancer’?. J. Exp. Clin. Cancer Res..

[34] Sakoda T., Kasahara N., Hamamori Y., Kedes L. (1999). A high-titer lentiviral production system mediates efficient transduction of differentiated cells including beating cardiac myocytes. J. Mol. Cell. Cardiol..

[35] Ma Y., Zhao M., Zhong J., Shi L., Luo Q., Liu J., Wang J., Yuan X., Huang C. (2010). Proteomic profiling of proteins associated with lymph node metastasis in colorectal cancer. J. Cell. Biochem..

[36] Zhong J., Zhao M., Ma Y., Luo Q., Liu J., Wang J., Wang J., Yuan X., Sang J., Huang C. (2012). UCHL1 acts as a colorectal cancer oncogene via activation of the β-catenin/TCF pathway through its deubiquitinating activity. Int. J. Mol. Med..

[37] Yu J., Cheng Y.Y., Tao Q., Cheung K.F., Lam C.N., Geng H., Tian L., Wong Y.P., Tong J.H.M., Ying J. (2009). Methylation of protocadherin 10, a novel tumor suppressor, is associated with poor prognosis in patients with gastric cancer. Gastroenterology.

