# Supplementary material for: Knocking down USP39 Inhibits the Growth and Metastasis of Non-Small-Cell Lung Cancer Cells through Activating the p53 Pathway
Source: Int J Mol Sci. 2020 Nov 25;21(23):8949. doi: 10.3390/ijms21238949 (PMC7728369; doi:10.3390/ijms21238949)
Supplement: Supplementary file 1 [file ijms-21-08949-s001.pdf]

Table S1. the correlation between expression of USP39 and clinicopathological features of lung cancer patients.

Variable	number	USP39 expression		positive rate	χ^2	*p* value
		-	+			
Age						
< 60	45	15	30	66.67%	4.3859	< 0.05
⩾ 60	32	5	27	84.37%		
Sex						
Female	36	9	27	75%	0.034	> 0.05
Male	41	11	30	73.1%		
Tumor histological type						
Lung adenocarcinoma	67	17	50	74.6%	19.311	< 0.05
Lung squamous cell cancer	2	0	2	100%		
Bronchioloalveolar carcinoma	8	3	5	62.5%		
Clinical stage						
I+II	37	10	27	72.9%	0.201	> 0.05
III	24	5	19	79.2%		
Lymph node invasion						
no	49	12	37	75.5%	0.154	> 0.05
yes	28	8	20	71.4%		

P values were calculated in SPSS online using a *chi-square test*. *P* values < 0.05 were considered to indicate statistical significance.

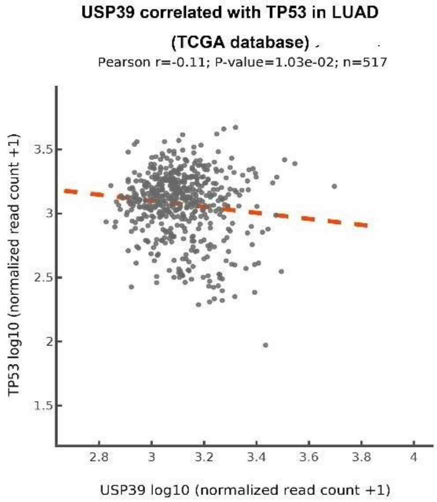

Figure S1. USP39 correlated with TP53 in lung adenocarcinoma (AIPuFu database).

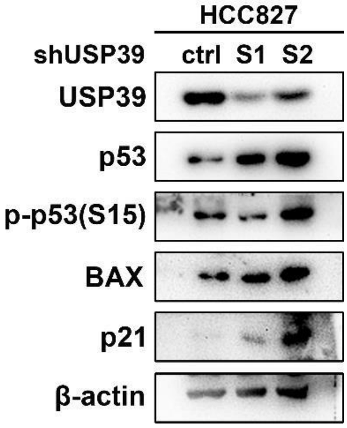

Figure S2. USP39 knockdown activates the p53 pathway. Western blot assay was carried out to examine the expression of p53, p-p53(S15), p21 and BAX in HCC827 cells.

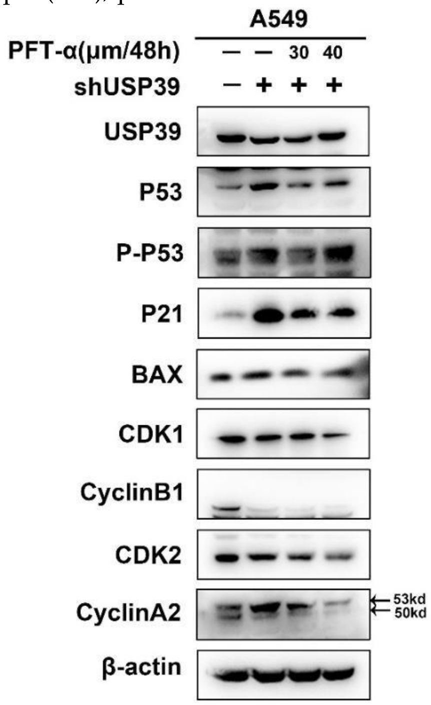

Figure S3. The effect of PFT-α on the p53 pathway activated by USP39 knockdown. Western blot assay was employed to examine the expression of p53, p-p53(S15), p21, BAX, CDK1, CyclinB1, CDK2

and CyclinA2 in A549 cells.
